# Sleep deprivation induces delayed regeneration of olfactory sensory neurons following injury

**DOI:** 10.3389/fnins.2022.1029279

**Published:** 2022-12-01

**Authors:** Bing Han, Shu Kikuta, Teru Kamogashira, Kenji Kondo, Tatsuya Yamasoba

**Affiliations:** Department of Otorhinolaryngology and Head and Neck Surgery, Graduate School of Medicine, The University of Tokyo, Tokyo, Japan

**Keywords:** olfactory sensory neuron, olfactory dysfunction, quinone dehydrogenase 1, sleep deprivation, circadian activity

## Abstract

The circadian system, which is essential for the alignment of sleep/wake cycles, modulates adult neurogenesis. The olfactory epithelium (OE) has the ability to generate new neurons throughout life. Loss of olfactory sensory neurons (OSNs) as a result of injury to the OE triggers the generation of new OSNs, which are incorporated into olfactory circuits to restore olfactory sensory perception. This regenerative potential means that it is likely that the OE is substantially affected by sleep deprivation (SD), although how this may occur remains unclear. The aim of this study is to address how SD affects the process of OSN regeneration following OE injury. Mice were subjected to SD for 2 weeks, which induced changes in circadian activity. This condition resulted in decreased activity during the night-time and increased activity during the daytime, and induced no histological changes in the OE. However, when subjected to SD during the regeneration process after OE injury, a significant decrease in the number of mature OSNs in the dorsomedial area of the OE, which is the only area containing neurons expressing NQO1 (quinone dehydrogenase 1), was observed compared to the NQO1-negative OE. Furthermore, a significant decrease in proliferating basal cells was observed in the NQO1-positive OE compared to the NQO1-negative OE, but no increase in apoptotic OSNs was observed. These results indicate that SD accompanied by disturbed circadian activity could induce structurally negative effects on OSN regeneration, preferentially in the dorsomedial area of the OE, and that this area-specific regeneration delay might involve the biological activity of NQO1.

## Introduction

The mammalian circadian system is essential for alignment of sleep/wake cycles to the 24 h day and for sleep quality (Mohawk et al., 2012). The circadian system controls rhythms in behavior, hormone secretion, and brain metabolism, and modulates the complex multistep process of adult neurogenesis, which is crucial for brain plasticity (Alhola and Polo-Kantola, 2007; Krause et al., 2017). Therefore, its disturbance involves a change in circadian behaviors (Grandin et al., 2006) and could suppress adult neurogenesis, resulting in difficulty in learning and memory (Keisler et al., 2007; Goel et al., 2009; Rasch and Born, 2013).

The olfactory epithelium (OE), inside the nasal cavity, comprises the olfactory sensory neurons (OSNs), which have a special ability to regenerate from progenitor cells throughout life (Mori and Sakano, 2011). Individual OSNs express a single functional allele of one odorant receptor (OR) gene from either the class I or class II repertoires, giving rise to two distinct OR-expressing OSN populations: class I and class II OSNs (Bozza et al., 2009). Class I OSNs are substantially distributed in the dorso-medial region of the OE, and selectively express nicotinamide adenine dinucleotide phosphate H (NADPH) quinone oxido-reductase 1 (NQO1), which is an intracellular enzyme involved in cell protection from natural and exogenous quinones (Gussing and Bohm, 2004). NQO1 is also involved in the regulation of mitotic progression by directly interacting with silent mating-type information regulation (sirt) members, which are a family of signaling proteins involved in metabolic regulation (Chang et al., 2009; Kim et al., 2018; Kang et al., 2018). Because sleep deprivation (SD) significantly downregulates some sirt members and delays neurogenesis in the hippocampus (Chang et al., 2009), regeneration of NQO1-positive OSNs (class I OSNs) that interact with the sirtuin family may likewise be delayed by SD. We thus hypothesized that NQO1-positive and –negative OEs may possess different cell kinetics and exhibit different regeneration processes under SD intervention.

The uninjured OE primarily contains mature OSNs, with a very low rate of OSN regeneration and relatively static cell dynamics (Benson et al., 1984; Farbman et al., 1988). Because of its anatomical location, the OE is directly exposed to environmental agents entering the nasal cavity, leaving OSNs prone to injury. Injury-induced loss of mature OSNs in the OE causes a prompt and massive regeneration of new OSNs through the proliferation and differentiation of progenitor cells (Kikuta et al., 2015).

Mice were subjected to SD over a 2-week period. In addition, the olfactotoxic drug, methimazole, was administered to selectively injure OSNs (Sakamoto et al., 2007). Using these combined methods, the cell dynamics of OSNs following injury were histologically examined under SD intervention.

We found that SD intervention induced a significant decrease in OSNs following injury in the dorsomedial area of the OE, as determined by expression of the NQO1. The NQO1-positive OE exhibited fewer proliferative basal cells and apoptotic OSNs compared to the NQO1-negative OE. These results indicate that SD induce structurally negative effects on the regenerative process, specifically in the dorsomedial area of the OE, and that area-specific injury is involved in the bioactivation of NQO1.

## Materials and methods

### Animals

In this study, 10-week-old male C57BL/6J mice were used (CLEA, Tokyo, Japan). The mice were housed at 22° with a 12-h light and 12-h dark cycle, and given adequate standard pellet food and tap water before all experiments started.

### Sleep deprivation

**FIGURE 1:**
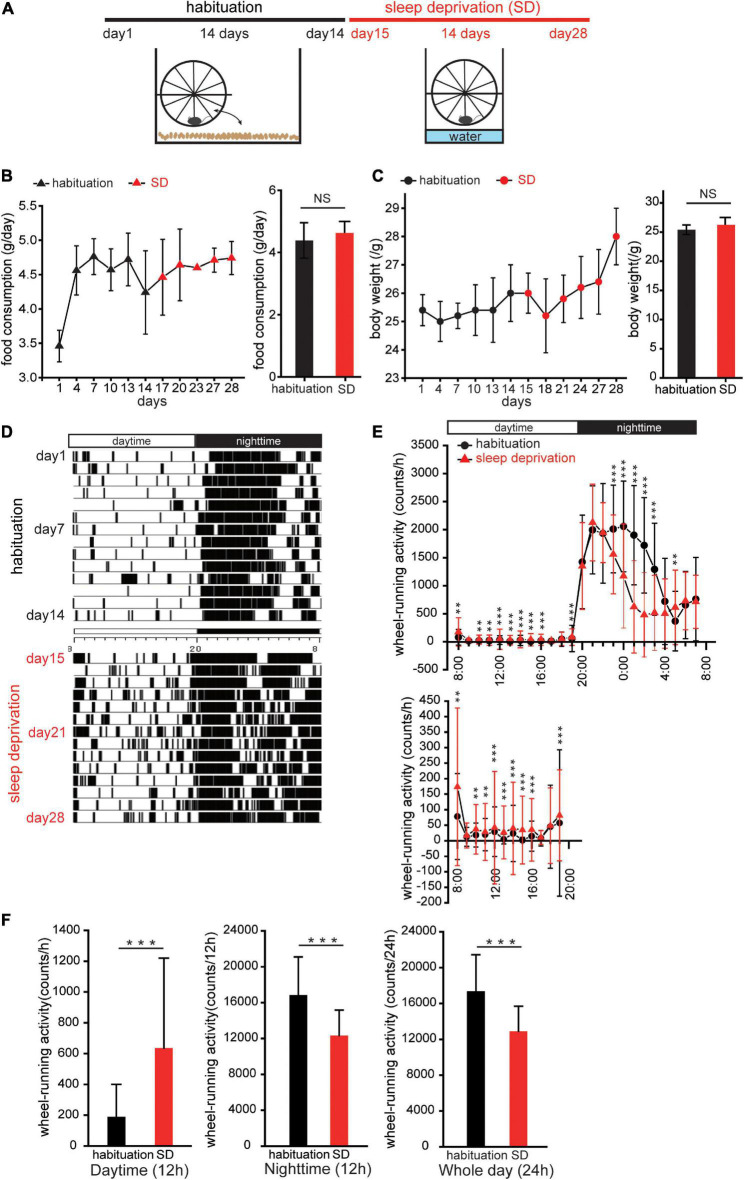
Sleep deprivation disturbs circadian activity. **(A)** Protocol for creating mice with disturbed circadian activity. Mice were kept in cages with running-wheels, which they could move freely onto and off in the first 2 weeks (habituation). In the third week, the bedding in each cage was replaced with water to a depth of 1.5 cm. Thereafter, the mice remained on the running-wheel throughout the day to avoid contact with the water (sleep deprivation, SD). **(B,C)** Food consumption and body weights during habituation and SD. Triangles (black, habituation; red, SD) represent changes in food consumption over 28 days; circles (black, habituation; red, SD) represent changes in body weights. No significant differences in food consumption and body weights between habituation and SD periods were observed (*n* = 5 mice; food consumption, *p* = 0.13; body weight, *p* = 0.06; Mann–Whitney *U* test). **(D)** Representative actogram of wheel-running events during habituation and SD periods. **(E)** Wheel-running activity during habituation (red, triangle) and SD (black, circle) periods plotted over 24 h. Wheel activity in 1 h bins was averaged for 14 days during habituation and SD periods, and the values from individual mice were averaged to yield a single 24 h profile (*n* = 5 mice). The lower figure shows wheel-running events during the daytime at different scales. *p < 0.05, **p < 0.01, and ***p < 0.001. **(F)** Comparison of wheel-running activity between habituation and SD periods during the daytime (left), night-time (middle), and whole day (right), respectively (*n* = 5 mice, ***p < 0.001; Mann–Whitney *U* test).

Mice with modulated circadian activity were created through a two-step process of habituation and SD. The purpose of the habituation period was to familiarize the mice with the wheel environment. During this period, male mice were individually maintained in plastic cages with running-wheels (SW-15S, Melquest, Toyama, Japan) and food access *ad libitum* for 2 weeks under the 12-h light and 12-h dark cycle (Figure 1A).

In the SD period, mice were exposed to a continuous stress imposed by the perpetual avoidance of water on a wheel (SW-15-SD, Melquset, Toyama, Japan) (Miyazaki et al., 2013). Briefly, paper-chip bedding was replaced with water to a depth of 1.5 cm. A running-wheel was mounted above the plastic cage, and the mice were forced to stay or run on a running-wheel constantly (Figure 1A). Wheel-running activity was continuously recorded at 1 min intervals using the Chronobiology Kit (Stanford Software Systems, Stanford, CA, USA), and activity data are displayed as actograms (Figure 1D). To observe the amount of wheel-running activity in a 24-h profile, the wheel activity of each mouse was accumulated in 1 h bins during the 2-week habituation and 2-week SD periods, and then averaged to yield a single 24 h profile (Figure 1E).

### Methimazole administration

Methimazole (40 mg/kg; Merck KGaA, Darmstadt, Germany) dissolved in saline was administered intraperitoneally 3 days before the end of the 2-week habituation period to ablate OSNs because the greatest loss of existing OSNs is observed at day 3 post-administration. All mice were sacrificed under deep anesthesia with a combined intraperitoneal injection of ketamine (100 mg/kg) and xylazine (9 mg/kg).

### Immunohistochemistry

Mice were perfused intracardially with 4% paraformaldehyde in 0.1 M phosphate buffer, decapitated, and postfixed for 24 h in the same fixative. The nasal tissues, including the OE, were decalcified with 10% ethylenediaminetetraacetic acid (EDTA) (pH 7.0) and embedded in paraffin. Coronal sections (4 μm thick) were cut and mounted onto silane-coated slides. The sections underwent immunofluorescence staining as previously described (Tuerdi et al., 2020). Briefly, following deparaffinization and rehydration, sections were immersed in Antigen Retrieval Solution (S1700; Dako Cytomation, Kyoto, Japan) and autoclaved at 121°C for 20 min to allow antigen retrieval to occur. Subsequently, sections were incubated for 20 min with a blocking solution containing 10% bovine albumin serum (Thermo Fisher Scientific, Fremont, CA, USA) to block the binding of non-specific antibodies.

Immunohistochemistry was performed with one or two of the following primary antibodies: anti-olfactory marker protein (OMP, goat polyclonal, 1:3000 dilution; Wako Chemicals, Richmond, VA, USA), anti-NQO1 (rabbit monoclonal, 1:500 dilution; Abcam, Cambridge, MA, USA), anti-sirt2 (rabbit monoclonal, 1:500 dilution; Abcam, Cambridge, MA, USA), anti-activated/cleaved caspase 3 (rabbit polyclonal, 1:500 dilution; Cell Signaling Technology, Danvers, MA, USA), and anti-ki67 (rabbit polyclonal, 1:500 dilution; Thermo Fisher Scientific). Secondary antibodies were donkey anti-goat Alexa Fluor 488 (1:1000; Invitrogen, Eugene, OR, USA) and donkey anti-rabbit Alexa Fluor 594 (1:1000; Invitrogen).

For NQO1 and sirt2 co-staining, a different protocol was required as both antibodies were raised in rabbit. Following deparaffinization, rehydration, antigen retrieval, and antigen blocking, as above, samples were incubated with anti-sirt2 (1:500; Abcam) antibody at 4° overnight. Subsequently, samples were incubated with both donkey anti-rabbit Alexa Fluor 488 at room temperature for 1 h. After washing with PBS several times, the sections were incubated with anti-NQO1 (1:500; Abcam) antibody at 4° overnight before incubation with donkey anti-rabbit Alexa Fluor 594.

### Analysis

For each OE, three coronal sections located between the caudal and the rostral OE regions were examined, and each section was cut at 500 μm intervals. The number of OSNs labeled by anti-OMP, anti-NQO1, anti-activated caspase-3, and anti-Ki67 antibodies was quantitatively analyzed using sections with single or double immunostaining for each antigen and counterstaining with DAPI. Any immunostaining that exceeded two standard deviations (SDs) of mean background intensity of the connective tissue under the lamina propria was considered positive. In each section, three OE areas (each, 100 μm) from both the left and right NQO1-positive OEs and two areas each from the left and right NQO1-negative OEs were arbitrarily selected, and the number of immunostained cells (OMP-, NQO1-, caspase- 3-, and ki67-positive cells) was measured. Data were quantified as the number of positive cells per 100 μm OE. All values are shown as mean ± SD, and the analysis for immunostained cells was performed using ImageJ software (NIH).

### Enzyme-linked immunosorbent assay

Blood cortisol level was determined using a Mouse DetectX Cortisol ELISA Kit (Arbor Assays, MI, USA), according to the manufacturer’s instructions. Blood samples were collected under deep anesthesia with a combined intraperitoneal injection of ketamine (100 mg/kg) and xylazine (9 mg/kg).

### Western blotting analysis

Olfactory mucosa was collected from normal, injury only, and injury + SD mice under deep anesthesia with a combined intraperitoneal injection of ketamine (100 mg/kg) and xylazine (9 mg/kg). OE protein purification was performed with a Nucleo Spin RNA/Protein purification kit according to the manufacturer’s instructions (NucleoSpin, Machrrey-Nagel GmbH & Co., Düren, Germany). Total proteins were quantified by using bicinchoninic acid (BCA) Protein Assay Kit (Pierce, Thermo Fisher Scientific, Fremont, CA, USA). And 30 μg of protein sample was loaded on a 10% SDS-PAGE gel (e-PAGEL HR, ATTO, Motoasakusa, Tokyo, Japan) along with a protein marker, separated at 135 V, 35 mA in MOPS SDS running buffer (Invitrogen, Fremont, CA, USA) for 80 min, and then electro-transferred (320 mA for 7 min) onto a polyvinylidene fluoride membrane (*Trans*-Blot Turbo Transfer System Transfer Pack, Bio-Rad Laboratories, CA, USA). Samples were blocked in 2% skim milk in Tris-buffered saline/Tween (TBS/T) buffer for 30 min at room temperature. The anti-sirt2 antibody (rabbit monoclonal, 1:1000 dilution; Abcam) was used to detect the sirt2 protein in the OE, and an anti-β-actin antibody (rabbit polyclonal, 1:2000 dilution; Medical & Biological laboratories Co., Tokyo, Japan) was used as an internal reference. Membranes were incubated with the anti-sirt2 antibody or anti-β-actin antibody overnight at 4°, washed several times in TBS/T buffer, and incubated with anti-rabbit horseradish peroxidase (HRP)-conjugated antibody (rabbit monoclonal, 1:3000 dilution; Cytiva, Tokyo, Japan) for 1 h at room temperature. All washes were performed in TBS/T for 4 × 5 min. The blots were imaged using the western blotting detection reagents (Amersham ECL Prime Western Blotting Detection Reagents, Cytiva).

### Statistical analyses

**FIGURE 2:**
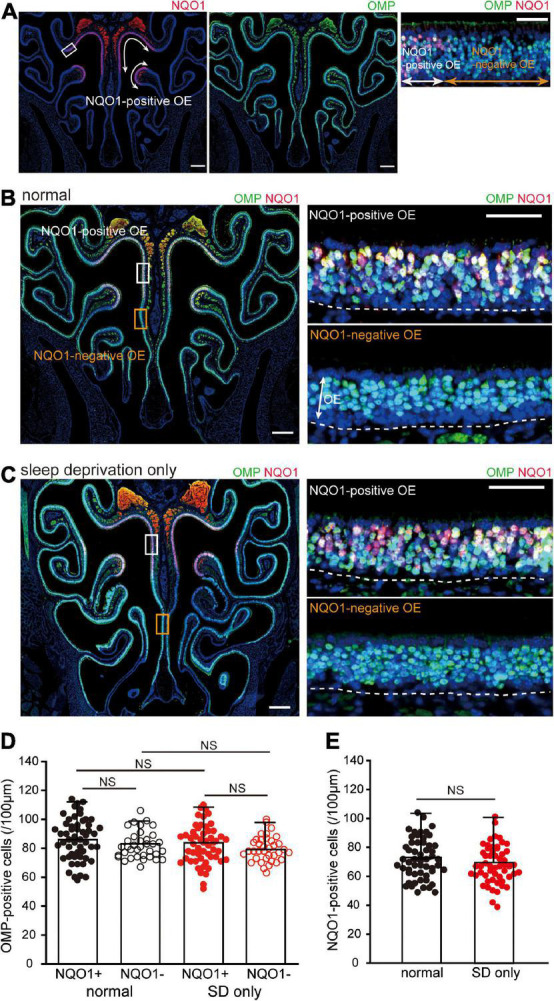
Sleep deprivation (SD) does not induce histological changes in the OE. **(A)** Photomicrographs of representative coronal sections from normal mice stained with anti-NADPH quinone oxido-reductase 1 (NQO1) antibody (left, red) and anti-olfactory marker protein (OMP) antibody (middle, green). Mature olfactory sensory neurons (OSNs) were evenly distributed within the OE, but NQO1-positive OSNs were confined to the upper nasal septum and upper concha bullosa, as indicated by the arrow range. Left and middle images, lower magnification; right image, higher magnification image of the area indicated by the square in the left image. Scale, 300 μm at low magnification; 50 μm at high magnification. **(B,C)** Photomicrographs of representative coronal sections from normal **(B)** and SD mice **(C)** stained with anti-OMP antibody (green) and anti-NQO1 antibody (red). Images on the right show higher magnification images captured from the areas indicated by the squares on the image on the left (upper, NQO1-positive OE; lower, NQO1-negative OE). The dashed line represents the basement membrane of the OE. Scale, 300 μm at low magnification; 50 μm at high magnification. OE, olfactory epithelium. **(D)** Counts of OMP-positive cells of NQO1-positive (NQO1+) and NQO1-negative (NQO1 –) OEs in normal and SD only mice (SD only). SD did not induce changes in the number of OMP-positive cells between the NQO1-positive and –negative OEs. NS, not significant; Steel–Dwass test. **(E)** Counts of NQO1-positive cells in normal (black) and SD only mice (red). Significant changes were not observed histologically between normal and SD only mice (NS, not significant, Mann–Whitney test).

**FIGURE 3:**
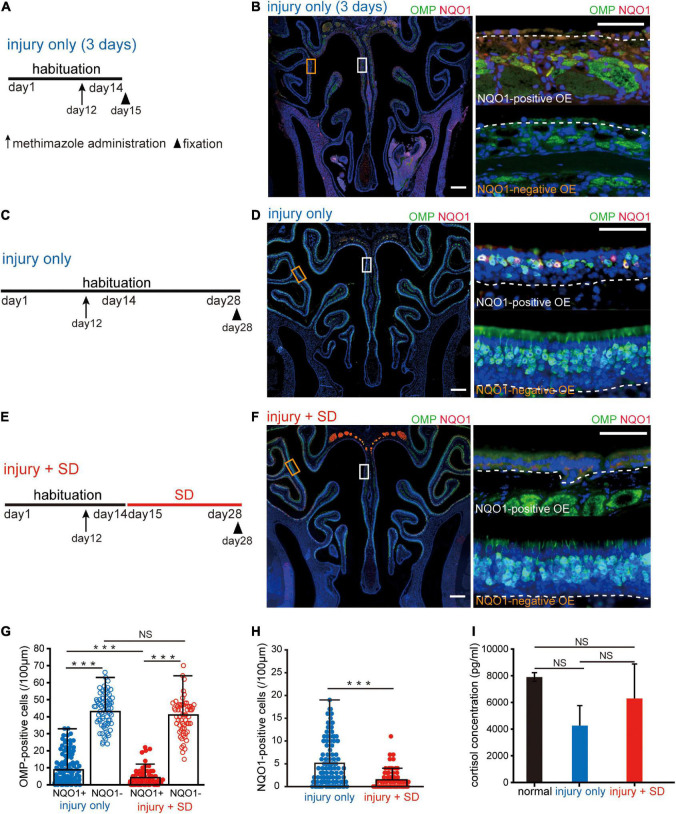
Short-term sleep deprivation selectively induces delayed regeneration in the NADPH quinone oxido-reductase 1 (NQO1)-positive olfactory epithelium (OE) following injury. **(A)** Time course and experimental design. Methimazole was administered on day 12, and perfusion with a fixative was conducted on day 15. Arrow indicates the timing of methimazole administration, and arrowhead indicates the timing of fixation. **(B)** Photomicrographs of representative coronal sections captured 3 days after methimazole administration (injury only, 3 days). Pictures on the right show higher magnification images captured from the areas indicated by the squares on the left picture (upper, NQO1-positive OE; lower, NQO1-negative OE). The dashed line represents the basement membrane of the OE. Scale: 300 μm at low magnification, 50 μm at high magnification. **(C)** Time course and experimental design. Methimazole was administered on day 12, and perfusion with a fixative was performed on day 28. **(D)** Photomicrographs of representative coronal sections at 16 days following methimazole administration (injury only). Pictures on the right show higher magnification images captured from the areas indicated by the squares on the left picture (upper, NQO1-positive OE; lower, NQO1-negative OE). Scale: 300 μm at low magnification, 50 μm at high magnification. **(E)** Time course and experimental design. Methimazole was administered at day 12 (arrow) after starting habituation. Three days after methimazole administration, a 2-week SD was initiated, and fixation was conducted on day 28 (arrowhead). **(F)** Photomicrographs of representative coronal sections following methimazole-induced injury and sleep deprivation (injury + SD). Pictures on the right show higher magnification images captured from the areas indicated by the squares on the left picture (upper, NQO1-positive OE; lower, NQO1-negative OE). Scale: 300 μm at low magnification, 50 μm at high magnification. **(G)** Counts of olfactory marker protein (OMP)-positive cells of NQO1-positive (NQO1+) and NQO1-negative (NQO1 –) OEs in injury only and injury + SD mice. In injury only mice, the number of OMP-positive olfactory sensory neurons (OSNs) in the NQO1-positive OE is significantly lower than in the NQO1-negative OE. In mice with injury combined with SD, the number of OMP-positive cells in the NQO1-positive OE was lower than that in the NQO1-negative OE. However, the degree of reduction was significantly greater in the NQO1-positive OE when mice were exposed to a 2-week SD compared with injury only mice (***p < 0.001; NS, not significant; Steel–Dwass test). **(H)** Counts of NQO1-positive cells in injury only and injury + SD mice. The number of the NQO1-positive OSNs in mice with injury combined with SD was significantly lower than the number of the NQO1-positive OSNs in injury only mice (***p < 0.001; Mann–Whitney *U* test). **(I)** Comparison of blood cortisol levels among normal, injury only, and injury + SD mice. No significant changes in cortisol levels were observed among normal, injury only, and injury + SD mice (NS, not significant, Steel–Dwass test).

**FIGURE 4:**
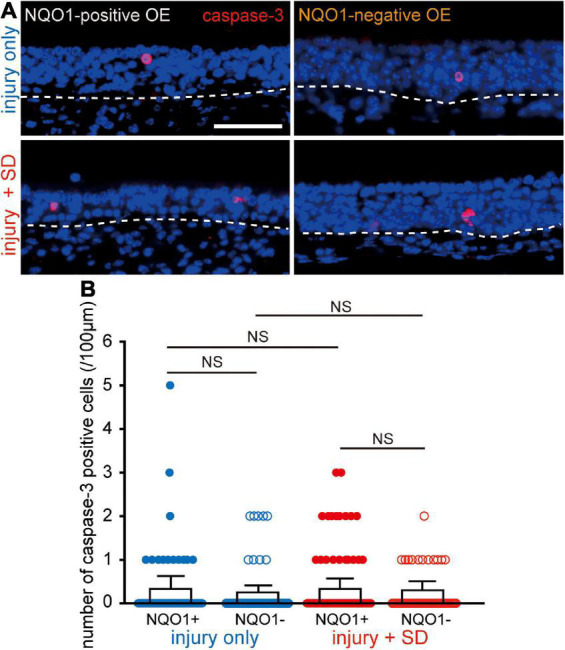
Neither injury only nor injury combined with sleep deprivation (SD) induces apoptosis. **(A)** Coronal sections stained with anti-caspase-3 antibody in the NQO1-positive (left), and –negative OE (right) in mice with injury only (upper, injury only) and mice with injury combined with SD (lower, injury + SD). The dashed line represents the basement membrane of the olfactory epithelium (OE). Scale, 50 μm. **(B)** Counts of cleaved caspase-3-positive cells in the NQO1-positive and –negative OE, in injury only and injury + SD mice. There was no significant change in the number of caspase-3-positive cells seen in the NQO1-positive OE in mice with injury combined with SD compared with that in mice with injury only. NS, not significant; Steel–Dwass test.

**FIGURE 5:**
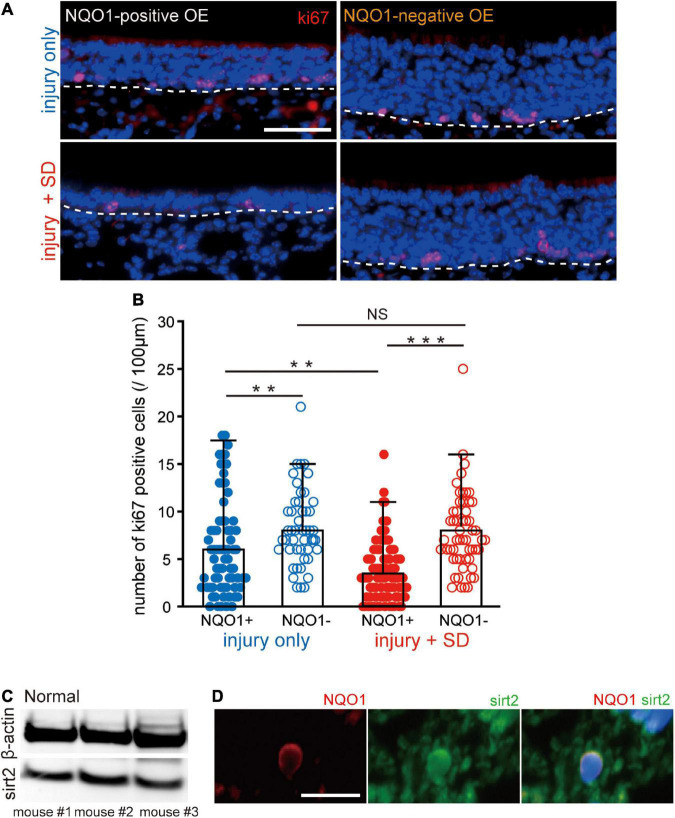
Sleep deprivation induces downregulation of proliferation in the NADPH quinone oxido-reductase 1 (NQO1)-positive olfactory epithelium (OE) following injury. **(A)** Coronal sections stained with anti-ki67 antibody in the NQO1-positive (left) and –negative OEs (right), in mice with injury only (upper, injury only) and mice with injury combined with sleep deprivation (SD) (lower, injury + SD). The dashed line represents the basement membrane of the OE. Scale, 50 μm. **(B)** Counts of ki67-positive cells in the NQO1-positive and –negative OE, in injury only and injury + SD mice. Injury only mice exhibited fewer ki67-positive cells in the NQO1-positive OE than in the NQO1-negative OE. In mice with injury combined with SD, the number of ki67-positive cells was significantly lower in the NQO1-positive OE compared with the NQO1-negative OE. The extent of the reduction in the NQO1-positive OE in injury + SD mice was greater than that in injury only mice. ***p < 0.001, **p < 0.05, NS, not significant, Steel–Dwass test. **(C)** Detection of sirt2 protein in the OE. Total protein from 30 μg weight olfactory mucosa was separated and analyzed for sirt2 and β-actin by western blotting. The anti-β-actin antibody was used as an internal reference. **(D)** Coronal sections stained with anti-NQO1 (red) antibody and anti-sirt2 (green) antibody in mice with injury combined with SD. NQO1-positive cell was co-stained with an anti-sirt2 antibody. Scale, 20 μm.

The data were statistically evaluated in Origin Pro software (Origin Lab Corporation, Northampton, MA, USA) and JMP Statistical Discovery software (SAS Institute Japan, Tokyo, Japan). Data in Figures 1B,C,E,F, 2E, 3H, were analyzed using the Mann–Whitney *U*, and the Steel–Dwass test was used to analyze data in Figures 2D, 3G,I, 4B, 5B. A *p*-value of < 0.05 was considered statistically significant.

### Study approval

All animal studies were approved by the Experimental Animal Research Committee at the University of Tokyo and carried out in accordance with the approved guidelines.

## Results

### Sleep deprivation disturbs circadian locomotor activity

Mice were kept in cages with running-wheels, which they could move freely onto and off, for a 2 week period to acclimatize animals to the running-wheel environment (habituation, Figure 1A; Miyazaki et al., 2013). In the third week, the bedding in each cage was replaced with water to a depth of 1.5 cm. Thereafter, the mice remained on the running-wheel constantly to avoid contact with the water [SD, Figure 1A]. This method easily allowed for a sustained stress load on the mice over a 2-week period. Food consumption and body weight changes were measured to determine whether stress-induced overeating or weight gain was present (Figures 1B,C). No significant changes in food consumption and body weight were observed in the SD period compared to the habituation period (body weight: *n* = 5 mice, *p* = 0.06; food consumption: *n* = 5 mice, *p* = 0.13, Mann–Whitney *U* test; Figures 1B,C). These results suggest that SD intervention does not induce body weight gain and potentially has no influence on energy metabolic systems.

Comparison of actograms of the habituation period and the SD period (Figures 1D,E, respectively) demonstrated that during the daytime of the SD period, wheel-running activity was typically higher than in the habituation period, while during the night-time, wheel-running activity was typically lower in the SD period than in the habituation period (*n* = 5 mice, ^**^p < 0.05, ^***^p < 0.001, Mann–Whitney *U* test, Figure 1E). When analyzed separately for the daytime and night-time, wheel-running activity during the daytime of the SD period was significantly greater than in the habituation period, while during the night-time, wheel-running activity was significantly lower in the SD period than in the habituation period (*n* = 5 mice, ^***^p < 0.001, Mann–Whitney *U* test, Figure 1F). For the entire day, daily (24 h) wheel-running activity during the SD period was significantly less than in the habituation period (*n* = 5 mice, ^***^p < 0.001, Mann–Whitney *U* test, Figure 1F). These results suggest that SD intervention disturbs circadian locomotor activities.

### Sleep deprivation for 2 weeks does not induce histological changes in the olfactory epithelium

We next examined the effect of SD on tissue homeostasis in the uninjured OE. When coronal sections of the OE were immunostained with anti-NQO1 antibody (Figure 2A, left) and anti-OMP antibody, to allow identification of mature OSNs (Figure 2A, middle), mature OSNs were evenly distributed across the OE, while NQO1-positive OSNs were limited to the upper nasal septum and upper concha bullosa (indicated by the arrow range in Figure 2A, left). Moreover, NQO1-positive and –negative OEs could be easily distinguished (Figure 2A, right).

When colocalization of OMP and NQO1 was compared in normal mice, no difference in the number of OMP-positive OSNs was seen between the NQO1-positive and –negative OE (*n* = 3 mice per group; normal: NQO1-positive OE vs. NQO1-negative OE, *p* = 0.11; Mann–Whitney *U* test; Figures 2B,D). Under SD intervention, there was no significant difference in the number of OMP-positive OSNs between the two different OEs (SD only: NQO1-positive OE vs. NQO1-negative OE, *p* = 0.65; Steel–Dwass test; Figures 2C,D). Furthermore, the number of OMP-positive OSNs in SD only mice was not significantly different from that in normal mice in both the NQO1-positive and –negative OEs [NQO1-positive (normal) vs. NQO1-positive (SD only), *p* = 0.48; NQO1-negative (normal) vs. NQO1-negative (SD only), *p* = 0.76; Steel–Dwass test; Figure 2D]. No significant differences were observed in NQO1-positive OSNs between normal and SD only mice (*n* = 3 mice per group; normal vs. SD only, *p* = 0.14; Mann–Whitney *U* test; Figure 2E). These results indicate that although the expression pattern of NQO1 is different, the number of mature OSNs in each OE is equally distributed, and their numbers are not affected by a 2-week SD intervention.

### Sleep deprivation for 2 weeks selectively induces delayed regeneration following injury in NADPH quinone oxido-reductase 1-positive olfactory epithelium

Next, we examined whether the NQO1-positive OE showed histological changes in response to SD intervention during the regeneration process following injury. During the first 2 weeks after OE injury, differentiation of basal progenitor cells is markedly enhanced to allow the number of OSNs to recover to pre-injury levels (Kikuta et al., 2015). We therefore focused on the regeneration of OSNs during the first 2 weeks following injury, when cellular dynamics are likely to be greatest.

Firstly, the OE was histologically evaluated 3 days after administration of the olfactory toxic drug methimazole [injury only (3 days), Figure 3A]. Analysis of coronal sections of the injured OE immunostained with anti-OMP and anti-NQO1 antibodies (Figure 3B) demonstrated that, with the exception of basal progenitor cells, almost all OSNs undergo cell death, and that both the NQO1-positive and –negative OE were uniformly injured (Figure 3B). These results suggest that the stage for differentiation of OSNs is reset 3 days after injury, providing an opportunity to study the kinetics of cells within the OE. We next examined the effects of SD on regeneration of OSNs following injury (Figure 3C). Figure 3D shows a representative coronal section of the OE immunostained with anti-OMP and anti-NQO1 antibodies 16 days following injury. In the NQO1-negative OE, the number of OSNs returned to pre-injury levels, but in the NQO1-positive OE, the OE was thinner and had fewer OSNs.

Next, mice were exposed to a 2-week SD at 3 days following injury (methimazole-induced injury plus SD; injury + SD, Figure 3E). In injury only mice, the number of OMP-positive OSNs in the NQO1-positive OE was significantly lower than in the NQO1-negative OE (*n* = 5 mice per group; injury only: NQO1-positive OE vs. NQO1-negative OE, ^***^p < 0.001; Steel–Dwass test; Figures 3F,G). These results suggest that the degree of regeneration following the injury is not uniform within the OE.

In mice with injury combined with SD, the number of OMP-positive OSNs in the NQO1-positive OE was significantly lower than that in the NQO1-negative OE (injury + SD: NQO1-positive OE vs. NQO1-negative OE, ^***^p < 0.001; Steel–Dwass test; Figure 3G). However, the degree of reduction was significantly greater in the NQO1-positive OE when mice were exposed to a 2-week SD [NQO1-positive (injury only) vs. NQO1-positive (injury + SD), ^***^p < 0.001; Steel–Dwass test; Figure 3G]. No significant difference in the number of OMP-positive cells was observed in the NQO1-negative OE between injury only mice and injury + SD mice [NQO1-negative (injury only) vs. NQO1-negative (injury + SD), *p* = 0.16; Steel–Dwass test; Figure 3G]. Furthermore, the number of the NQO1-positive OSNs was significantly lower in mice with injury combined with SD than in injury only mice (*n* = 5 mice per group; injury only vs. injury + SD; ^***^p < 0.001; Mann–Whitney *U* test; Figure 3H). These results suggest that SD intervention accompanied by the disturbed circadian locomotor activities enhances the delay in regeneration following injury, but the effect is limited to a specific area within the OE.

Various negative regulators of regeneration could be affected by stress, and SD is often perceived as a physiological stressor (Inoué et al., 1995). Thus, the effect of SD on regeneration following injury in the NQO1-positive OE may be caused by excessive stress. We therefore assessed the degree of stress by measuring blood cortisol levels. No significant changes in cortisol levels were observed among normal, injury only, and injury + SD mice (*n* = 5 mice per group; normal vs. injury only, *p* = 0.19; injury only vs. injury + SD, *p* = 0.55; normal vs. injury + SD, *p* = 0.98; Steel–Dwass test; Figure 3I). These results indicate that the reduction in the number of OMP-positive OSNs in the NQO1-positive OE was indeed due to an effect of SD intervention, rather than excessive stress associated with experimental manipulation.

### Sleep deprivation does not increase apoptotic olfactory sensory neurons in the NADPH quinone oxido-reductase 1-positive olfactory epithelium

The reduction in the number of OSNs in the NQO1-positive OE by intervening SD may be due either to an increase in OSN cell death, a decrease in the number of newly generated OSNs, or both. To examine these possibilities, we determined the expression of active caspase-3 in the OE under the two different experimental conditions (injury only, and injury + SD; Figure 4) using an antibody specific for the active (cleaved) form of caspase-3, a marker of apoptotic cell death that functions in most downstream caspase-activation cascades (Yuan et al., 2003).

Figure 4A shows representative images stained with anti-caspase-3 antibody of mice with injury only (upper, injury only) and mice with injury combined with SD (lower, injury + SD), in the NQO1-positive and –negative OEs (left, NQO1-positive OE; right, NQO1-negative OE). In injury only mice, there was no change in the number of caspase-3-positive cells between NQO1-positive and NQO1-negative OEs (*n* = 5 mice per group; injury only: NQO1-positive OE vs. NQO1-negative OE, *p* = 0.99; Steel–Dwass test; Figure 4B). In mice with injury combined with SD, a similar trend between the NQO1-positive and NQO1-negative OE was also observed (injury + SD: NQO1-positive OE vs. NQO1-negative OE, *p* = 0.93; Steel–Dwass test; Figure 4B). Furthermore, the number of caspase-3-positive cells in the NQO1-positive OE in mice with injury combined with SD was not significantly higher than in mice with injury only [NQO1-positive (injury only) vs. NQO1-positive (injury + SD), *p* = 0.46; Steel–Dwass test; Figure 4B], and similar trends were observed in the NQO1-negative OE [NQO1-negative (injury only) vs. NQO1-negative (injury + SD), *p* = 0.96; Steel–Dwass test; Figure 4B]. These results argue against the hypothesis of upregulation of cell death in the NQO1-positive OE.

### Sleep deprivation induces downregulation of proliferation in olfactory sensory neurons in NADPH quinone oxido-reductase 1-positive olfactory epithelium

The delayed regeneration observed in the NQO1-positive OE may result from decreased OSN proliferation. To examine the extent of OSN proliferation, we examined expression of the cell proliferation marker, ki67, in the NQO1-positive and –negative OEs (Figure 5). Figure 5A shows representative images stained with anti-ki67 antibody of sections obtained from mice with injury only (upper, injury only) and mice with injury combined with SD (lower, injury + SD) in the NQO1-positive and –negative OEs (left, NQO1-positive OE; right, NQO1-negative OE). In injury only mice, the proportion of ki67-positive cells in the NQO1-positive OE was significantly lower than in the NQO1-negative OE (*n* = 5 mice per group; injury only: NQO1-positive OE vs. NQO1-negative OE, ^**^p < 0.05; Steel–Dwass test; Figure 5B). Similarly, in mice with injury combined with SD, there were fewer ki67-positive cells in the NQO1-positive OE than in the NQO1-negative OE (injury + SD: NQO1-positive OE vs. NQO1-negative OE, ^***^p < 0.001; Steel–Dwass test; Figure 5B). Furthermore, the extent of the reduction in injury + SD mice was greater than in injury only mice [NQO1-positive (injury only) vs. NQO1-positive (injury + SD), ^**^p < 0.05; Steel–Dwass test; Figure 5B]. By contrast, no significant difference in the number of ki67-positive cells of the NQO1-negative OE was observed between injury only mice and injury + SD mice [NQO1-negative (injury only) vs. NQO1-negative (injury + SD), *p* = 0.90; Steel–Dwass test; Figure 5B]. Taken together, these results suggest that SD intervention does not increase apoptotic OSNs in the NQO1-positive OE, but instead leads to delayed regeneration due to a marked reduction in mitosis in progenitor basal cells in the NQO1-positive OE.

NADPH quinone oxido-reductase 1 regulates mitotic progression through modulation of sirt2 activity in the hippocampus (Chang et al., 2009; Kim et al., 2018). We therefore examined whether sirt2 is also expressed in the OE. As expected, western blot analysis demonstrated that sirt2 was expressed in the OE of three mice (Figure 5C). Furthermore, immunofluorescence staining demonstrated that sirt2 was co-expressed with NQO1 in OSNs (Figure 5D). These results suggest that the NQO1 expressed in the OE may regulate mitotic progression through interaction with sirt2.

## Discussion

In this study, we have examined how SD intervention affects the maturation of new OSNs following methimazole-induced injury. This method also necessarily involves some stress load. However, the method used in this study was found not to be an excessive stress load that would be accompanied by an increase in cortisol. SD mice demonstrated decreased locomotor activity during the night-time and increased activity during the daytime. Changes in circadian activity for 2 weeks did not cause histological changes in the OE. However, during the repair process following chemically induced OE injury, changes in circadian activity delay OSN regeneration, selectively in the NQO1-positive OE, which represents proliferating cells. Furthermore, in this region, while there was a decrease in the number of proliferative basal cells, no changes in apoptotic OSNs were observed. These results indicate that circadian activities play an important role in regenerative process preferentially in the NQO1-positive OE, and that their disturbance causes a delay in the incorporation of new OSNs into the neural circuit.

Olfactory sensory input is an important factor involved in regeneration of the OE. After methimazole-induced damage to the mouse OE, when olfactory input is blocked by silicon tube insertion, new OSNs fail to mature and instead undergo apoptosis (Kikuta et al., 2015). Insulin signaling is also a necessary factor for tissue regeneration. In diabetic mice, decreased insulin signaling prevents new OSNs from differentiating into mature OSNs, resulting in reduced olfactory function (Kuboki et al., 2021). In addition to these factors, sleep could play an important role in regeneration for some areas of the OE, and ensuring sleep without circadian rhythm disruption may be one of the factors necessary for functional recovery in the olfactory system.

Sleep disorder is defined by irregular circadian rhythm including difficulties in falling and/or staying asleep, followed by functional impairment while awake. We created SD mice under a weak stress continuously imposed throughout the day and night by the perpetual avoidance of water on a wheel to induce disturbed circadian locomotor activity, based on a previously reported technique (Miyazaki et al., 2013). Mouse models of sleep disorders have historically been based on SD techniques. Typical methods of inducing chronic SD include the classic disk-over-water technique and the use of slowly rotating wheel (Lopez-Rodriguez et al., 2004; Kim et al., 2007). These methods force mice to remain awake, and thus cannot be maintained for periods of 48 h or repeated for 20 h/day for 5 days because mice survivability decreases (Lopez-Rodriguez et al., 2004; Kim et al., 2007). Temporal exposure to conventional stressors, such as immobilization or electric shock at fixed times of the day, affects sleep time and consolidation (Pawlyk et al., 2008), while the timing of stress load provides mice with zeitgeber cues that induce stress anticipation activity (Barnum et al., 2007). Furthermore, in these model mice, 6–8 weeks of stress exposure gradually reduces gross and nocturnal locomotor activity, but does not affect diurnal locomotor activity. Thus, it does not affect circadian locomotor activity throughout the day and night. An environment in which the animal is placed in the territory of a male rat of the same species for an hour, which is social defeat stress, also rapidly suppresses nocturnal locomotor activity, but does not increase daytime activity (Meerlo et al., 1996).

One drawback of the SD method used here is its lack of versatility, as only a limited number of mouse strains have been examined for its effects on autonomic nervous system and hormone secretion (Miyazaki et al., 2013). In addition, this method uses the hedonistic nature of wheel running, a rewarding activity. Thus, it may be difficult to sustain a disturbance in circadian locomotor activity over a long period of time, as the mouse may become less hedonistic without appropriate rest.

In the C57BL/6 mice used in this study, the 2-week wheel environment did not cause changes in diet or body weight, but there are inter-strain differences in response to changes in environmental conditions. For example, in C3H/He mice, during 2 weeks of SD, food intake increases but body weight gradually decreases (Miyazaki et al., 2013). In general, C3H/He mice tend to exhibit more anxiety, depression-like behaviors, and stress responsivity compared with C57 BL/6 mice (Kopp et al., 1999). These differences in behavior and autonomic responses among mouse strains may have different effects on OE regeneration as well as changes in body weight and food intake after 2 weeks of SD.

The negative effects of continuous stress load have been hypothesized to be related to the cellular consequences of prolonged waking, including cell toxicity by excess glutamate release, free radicals, or elevated glucocorticoids, all of which may affect cell kinetics in the OE (Inoué et al., 1995; Mamelak, 1997; Guzmán-Marín et al., 2003). However, a previous report indicates that SD within 14 days does not increase apoptotic cell death, as determined by TUNEL in many brain regions (Cirelli et al., 1999). Similarly, we failed to observe a significant increase in active caspase-positive cells in the NQO1-positive OE compared to the NQO1-negative OE. Furthermore, we observed no significant increase in blood corticosterone levels under SD intervention, indicating that it is unlikely that the delayed regeneration in the NQO1-positive OE observed in mice with injury combined with SD is primarily due to excessive stress load. This suggests that other factors may be responsible for the adverse effects of SD.

Cell proliferation is regulated by several factors, including endogenous substances and inflammatory cytokines (Gould et al., 1999; Tanapat et al., 1999; Cameron and McKay, 2001; Kempermann, 2002). These factors are substantially affected by SD, and may provide a link between insufficient sleep and reduced neurogenesis. For instance, insulin-like growth factor, (IGF)-1, is one of several growth factors known as neurogenesis promoters (Trejo et al., 2001), but long-lasting SD in rats resulted in lower IGF-1 binding (Everson and Crowley, 2004). The brain-derived neurotrophic factor (BDNF) facilitates hippocampal neurogenesis (Scharfman et al., 2005; Guzman-Marin et al., 2006), and the hippocampal expression of BDNF was decreased after 48 h of SD (Guzman-Marin et al., 2006). Furthermore, exposure to IL-6 and TNF-a, which are increased after chronic SD, diminishes cell proliferation *in vitro* (Irwin et al., 2006; Haack et al., 2007). Thus, these factors may be one reason for delayed regeneration across the OE. However, they cannot explain the heterogeneous regeneration mechanisms observed within the OE such that SD delays regeneration of the NQO1-positive OE.

NADPH quinone oxido-reductase 1 plays multiple physiological roles as a consequence of its ability to directly interact with the sirt family members (Gaikwad et al., 2001; Kang et al., 2018). Indeed, NQO1 regulates mitotic progression by functioning as an NAD(P)H dehydrogenase through modulating sirt2 activity (Chang et al., 2009; Kim et al., 2018). Consistently, we observed coexpression of NQO1 and sirt2 in OSNs. The response to mitotic stress also involves sirt1, through its binding to NQO1 and its activation (Chang et al., 2009). Thus, the NQO1-positive OE could possess a cell proliferation mechanism that strongly depends on the interaction of NQO1 with sirt family members, suggesting that some sirt family members are downregulated under SD accompanied by disturbed circadian activity, selectively delaying regeneration of NQO1-positive OE.

A possible reason for delayed regeneration in the NQO1-positive OE in the absence of SD may be related to the different expression patterns of all-*trans*-retinoic acid (RA) in horizontal basal cells (HBC), one of the neurogenic stem cells. RA is a morphogen derived from Vitamin A, which regulates organogenesis and tissue regeneration, and is widely used to induce differentiation of pluripotent stem cell cultures (Gudas and Wagner, 2011). HBCs in the NQO1-positive OE differ significantly from those in the NQO1-negative OE with regard to RA availability (Login et al., 2015). RA bioavailability in NQO1-positive HBCs is low compared with NQO1-negative HBCs because of increased expression of the RA-degrading enzyme, cytochrome P450 family 26 (Håglin et al., 2020). Thus, NQO1-positive HBCs following injury may exhibit lower proliferative potential than NQO1-negative HBCs, even without SD intervention.

Although olfactory disorders have a wide variety of causes (Jones and Rog, 1998; Murphy et al., 2002; Brämerson et al., 2004), treatments to achieve complete tissue regeneration and functional recovery have not been well established. It is interesting from a therapeutic perspective to speculate that SD triggers reduced expression of the sirt family in the OE. Initial drug development efforts focused on sirt1 and sirt2 have yielded several promising activators that have now been through the first clinical trials, with evidence of safety and efficacy (Dai et al., 2018). Thus, sirt family members may become an attractive therapeutic target, potentially leading to the development of new agents for loss of OSNs in the NQO1-positive OE.

The axons of class I OSNs (NQO1-positive OSNs) are converged onto the dorsomedial and anteromedial region of the olfactory bulb (OB) (a dorsal domain for class I odorant receptors, D_I_ domain), while the axons of class II OSNs (NQO1-negative OSNs) are converged onto the dorsolateral, anterolateral (a dorsal domain for class II odorant receptors, D_II_ domain), and ventral region of the OB (a ventral domain for class II odorant receptors, V domain) (Mori and Sakano, 2011). This class-specific anatomical domain organization in the OB correlates with functional odor-induced innate responses (Mori and Sakano, 2011). The projection of class I OSNs to the D_I_ domain is responsible for innate aversive behavior to odorants produced from the spoiled foods, while the projection of class II OSNs to the D_II_ domain is responsible for innate fear responses to predator odors (Kobayakawa et al., 2007; Mori and Sakano, 2011). Furthermore, it has been reported that olfactory behaviors are altered by the biased OR class choice of OSNs (Enomoto et al., 2019). Class II-dominant mice created by genetic manipulation of the transcription factor, Bcl11b, exhibited the same reduction in aversion to odorants produced from spoiled foods and predator odors as the wild type, indicating that behavioral outputs toward two distinct aversive odorants depend on the populations of class I and class II OSNs. In wild mice, an equilibrium between the proportion of class I and class II OSNs allows for high sensitivity and subsequent appropriate reactions to danger-signaling odors, while an imbalance in the proportion of class I and II OSNs could have serious negative consequences for adaptation to the external environment and survival of the species.

There is increasing evidence that the circadian system regulates the multi-step process of adult neurogenesis through rhythmic systemic factors such as neurotransmitters, hormones, and intrinsic factors such as redox status and clock genes/molecular clocks within neural progenitor cells (Ali and von Gall, 2022). SD accompanied by disturbed circadian activity have been suggested to be a potential risk factor for the development of Alzheimer’s disease and related dementias and Parkinson’s disease, in addition to being a symptom of neurodegeneration (Leng et al., 2019). However the mechanistic link between circadian rhythms and neurodegeneration is still not fully understood. Therefore, a better understanding of how circadian activity modulates adult neurogenesis in various brain regions including the OE may provide a key to elucidate the pathophysiology of neurodegenerative diseases as well as olfactory disorders.

## Data availability statement

The raw data supporting the conclusions of this article will be made available by the authors, without undue reservation.

## Ethics statement

The animal study was reviewed and approved by the Experimental Animal Research Committee at the University of Tokyo.

## Author contributions

BH and SK designed the studies, wrote the manuscript, and created all drawings in figures. BH, SK, and TK performed the experiments. BH analyzed the data. BH, SK, TK, KK, and TY revised and finalized the manuscript. All the authors read and approved the manuscript.
